# A Dexterous, Glove-Based Teleoperable Low-Power Soft Robotic Arm for Delicate Deep-Sea Biological Exploration

**DOI:** 10.1038/s41598-018-33138-y

**Published:** 2018-10-03

**Authors:** Brennan T. Phillips, Kaitlyn P. Becker, Shunichi Kurumaya, Kevin C. Galloway, Griffin Whittredge, Daniel M. Vogt, Clark B. Teeple, Michelle H. Rosen, Vincent A. Pieribone, David F. Gruber, Robert J. Wood

**Affiliations:** 1 Department of Ocean Engineering, University of Rhode Island, Narragansett, RI USA; 2 School of Engineering and Applied Sciences, Harvard University, Cambridge, MA USA; 3 Wyss Institute for Biologically Inspired Engineering, Boston, MA USA; 4 Department of Mechanical Engineering, Tokyo Institute of Technology, Tokyo, Japan; 5 School of Engineering, Vanderbilt University, Nashville, TN USA; 6 The John B. Pierce Laboratory, Inc., New Haven, CT USA; 7 Cellular and Molecular Physiology and Department of Neuroscience, Yale University School of Medicine, New Haven, CT USA; 8 OceanX, Westport, CT USA; 9 Department of Natural Sciences, Baruch College, City University of New York, New York, NY USA; 10 PhD Progam in Biology, The Graduate Center City University of New York, New York, NY USA; 11 Radcliffe Institute for Advanced Study, Harvard University, Boston, MA USA

## Abstract

Modern marine biologists seeking to study or interact with deep-sea organisms are confronted with few options beyond industrial robotic arms, claws, and suction samplers. This limits biological interactions to a subset of “rugged” and mostly immotile fauna. As the deep sea is one of the most biologically diverse and least studied ecosystems on the planet, there is much room for innovation in facilitating delicate interactions with a multitude of organisms. The biodiversity and physiology of shallow marine systems, such as coral reefs, are common study targets due to the easier nature of access; SCUBA diving allows for *in situ* delicate human interactions. Beyond the range of technical SCUBA (~150 m), the ability to achieve the same level of human dexterity using robotic systems becomes critically important. The deep ocean is navigated primarily by manned submersibles or remotely operated vehicles, which currently offer few options for delicate manipulation. Here we present results in developing a soft robotic manipulator for deep-sea biological sampling. This low-power glove-controlled soft robot was designed with the future marine biologist in mind, where science can be conducted at a comparable or better means than via a human diver and at depths well beyond the limits of SCUBA. The technology relies on compliant materials that are matched with the soft and fragile nature of marine organisms, and uses seawater as the working fluid. Actuators are driven by a custom proportional-control hydraulic engine that requires less than 50 W of electrical power, making it suitable for battery-powered operation. A wearable glove master allows for intuitive control of the arm. The manipulator system has been successfully operated in depths exceeding 2300 m (3500 psi) and has been field-tested onboard a manned submersible and unmanned remotely operated vehicles. The design, development, testing, and field trials of the soft manipulator is placed in context with existing systems and we offer suggestions for future work based on these findings.

## Introduction

Since their introduction in the late 1950’s, manipulator systems developed for use in the harsh marine environment have focused on industrial and commercial applications to guide their design. Oceanographic engineers first modified nuclear reactor “hot work” manipulators to operate in the deep-sea^1,2^, and cross-industry collaboration continued throughout the 1980’s^3^. The major industry involved in deep-sea exploration and technology development during this era was the offshore oil and gas industry; demanding tasks required robust equipment, and as a result the majority of underwater manipulators have become sophisticated, heavy-duty systems^4^. Such arms are driven using energy-hungry hydraulic power systems, which directly affect the scale, design architecture, and operational time of deep-sea vehicles.

Deep-sea biologists are increasingly focused on conducting nuanced tasks associated with sampling, observing, and experimenting with fragile organisms^5^. This requirement has inspired a new field of marine robotics that is focused on delicate and precise manipulation, enabled in large part by the growing discipline of soft robotics^6^. These efforts fall into two categories: gripping devices, which have demonstrated functionality at a range of depths^7–13^, and full manipulators, which have only been demonstrated in shallow waters^14–16^. These new devices present an additional benefit in the form of power savings; they generally operate at dramatically reduced hydraulic pressures and/or utilize relatively weak electromechanical actuators, offering myriad benefits to deep-sea vehicle design and functionality.

**Figure 1:**
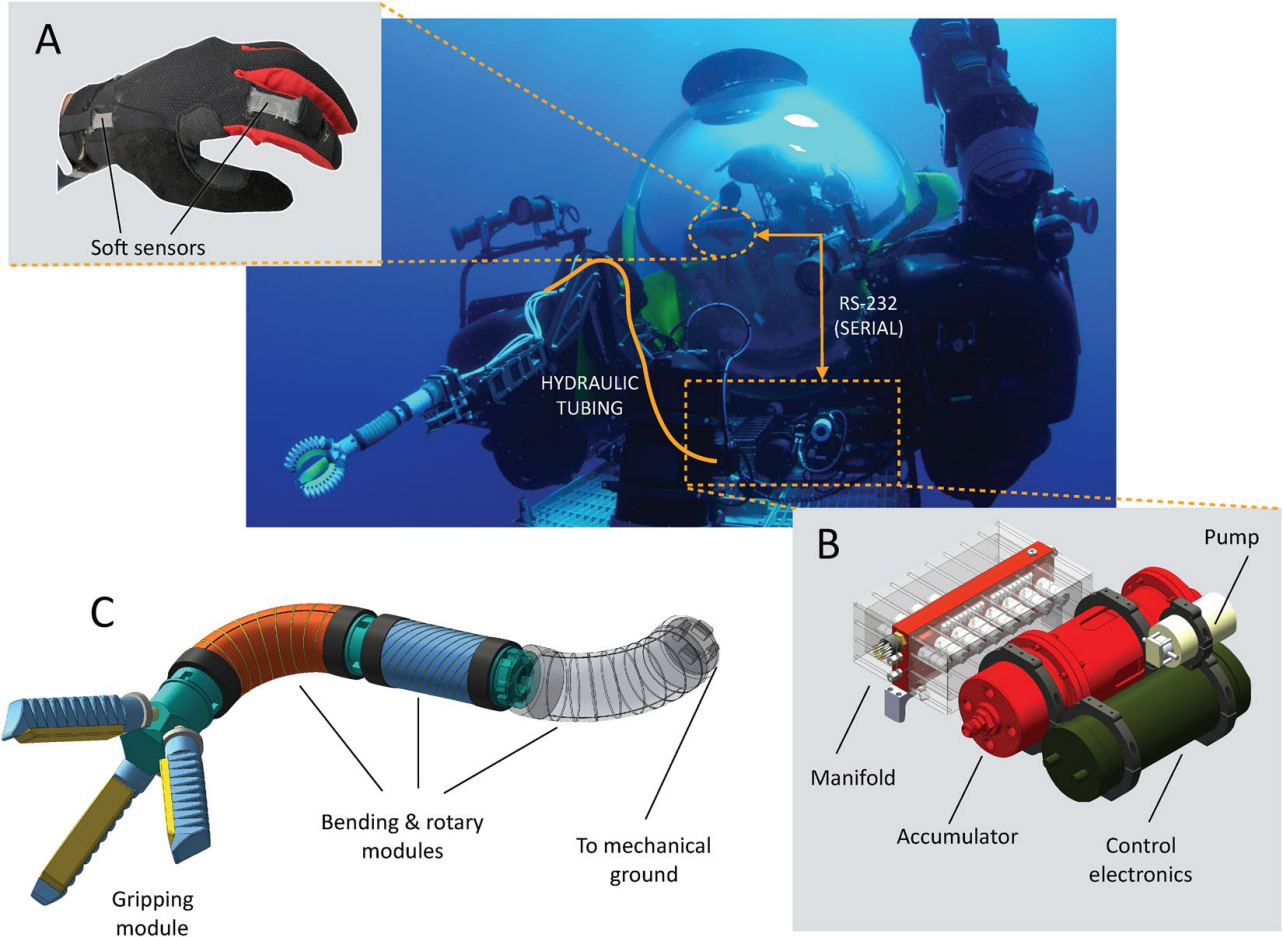
Overview of the deep-sea soft robotic arm system. (**A**) Control of actuators is achieved using a sensorized wireless glove, which coordinates the control of independent proportional valves that distribute pressure to the arm and end-effector actuators. (**B**) A custom open-circuit seawater engine regulates hydraulic pressure to independent ports, and can operate at depths of at least 2500 m. (**C**) The soft arm, consisting of bending, rotary, and gripping modules, can be mounted independently or as part of an existing manipulator system.

We present the first stand-alone soft robotic manipulator system designed for use in the deep-sea (Fig. 1). Our multi-degree-of-freedom arm consists of modular bending, twisting, and gripping modules which are powered using low-pressure seawater drawn from the surrounding environment. A novel hydraulic engine requiring less than 50 W of power to operate is also described. Teleoperation of the arm is achieved using a wireless glove outfitted with flexible soft sensors^17^. The manipulator system has demonstrated successful operation at hydrostatic pressures equivalent to a seawater depth of 2300 m, and has been field-tested onboard a manned submersible and an unmanned remotely operated vehicle.

## Results

**Figure 2:**
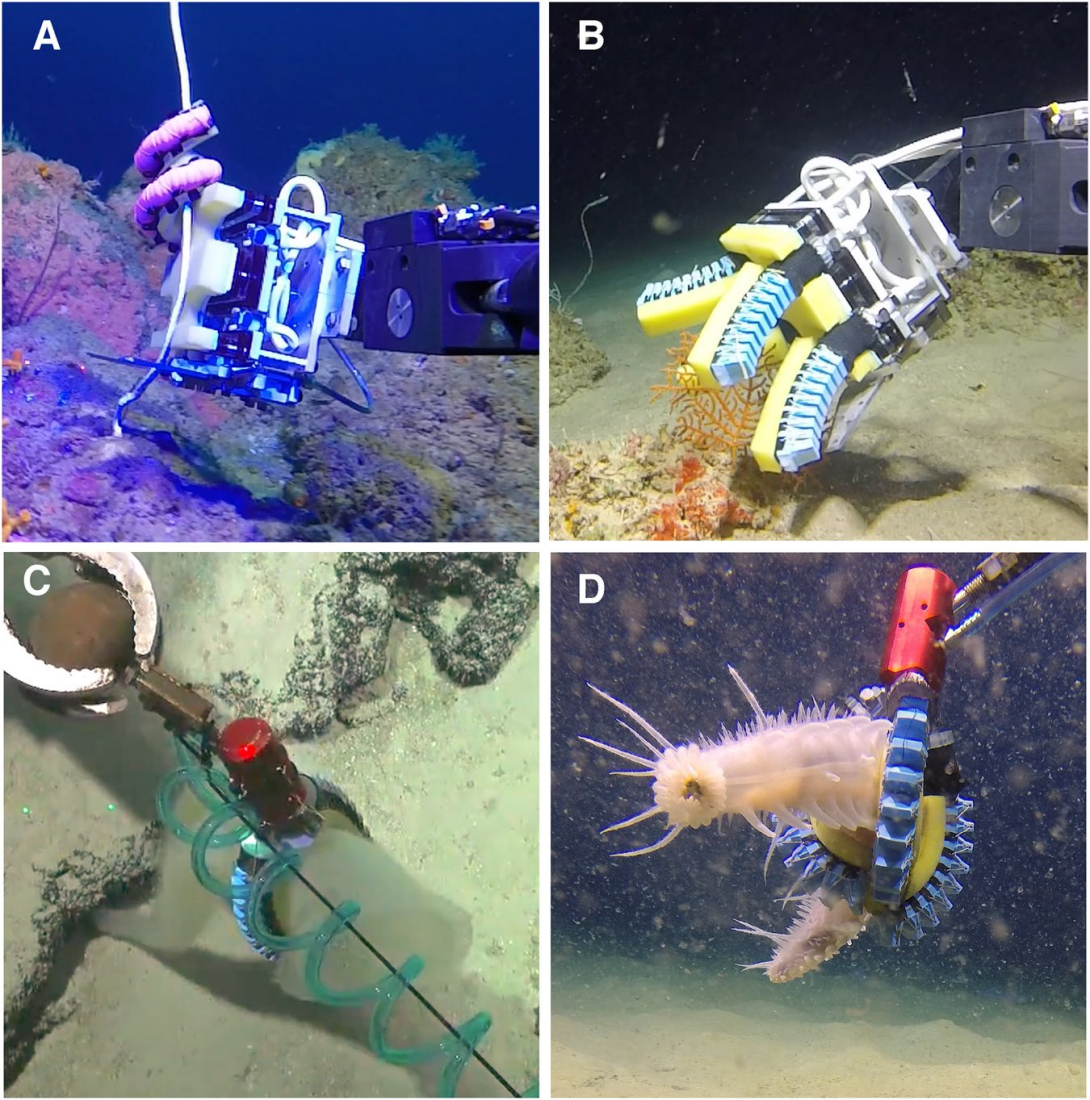
Examples of prior work on versatile soft grippers for deep-sea biological sampling. (**A**) “Boa” style fiber-reinforced actuator grasping a whip coral, and (**B**) four finger bellows-style actuator grasping a brittle scleractinian coral at 100 m in the Gulf of Eilat, Israel^10^. (**C**) A two finger bellows actuator grasping a glass sponge at 300 m depth at Carandolet Reef, Phoenix Islands^13^. Green laser dots at left side of image are 10 cm apart. (**D**) Three finger gripper with bellows-type actuators, grasping a holothurian at 1800 m in the Channel Islands National Marine Sanctuary, California.

The deep-sea remains the least explored biome on the planet^18^. Relatively robust animals such as corals and holothurians can be easily damaged by traditional heavy-duty manipulator systems, while gelatinous animals (with members spanning a diverse suite of fauna such as cnidarians, ctenophores, annelids, molluscs, chaetognaths, pelagic tunicates and appedicularians) often remain undescribed entirely because they are too fragile for successful collection via suction sampling^19^. Midwater sampling of such delicate-tissue animals is currently achieved using a jar-like device called a “D-sampler”, which is not ideally configured for seafloor sampling and is usually positioned by moving the entire submersible or ROV^20^. Soft robots are especially well suited to address these challenging specimen collections due to their potential for compliance matching^21^, and we improve the capacity of our first-generation grippers^10^, shown in Fig. 2, by eliminating their reliance on standard heavy-duty manipulators for positioning. This was accomplished by developing modular soft actuators that operate at similar pressure and flow rates (compared with the previous soft grippers) to compose a soft arm, along with a drive system cable of controlling them, thereby extending the capabilities of soft robotic grippers to conduct delicate deep-sea sampling.

### Modular soft manipulator

Traditional rigid-link manipulator systems rely on individual prismatic and rotary actuators to achieve multiple degrees of freedom. Alternative approaches to manipulation using soft and compliant materials have focused almost exclusively on continuum motion, resulting in a wide range of tentacle-like concepts^22^. Modularity presents an advantage for these soft arms, allowing for discrete control of actuators that individually can achieve more complex motions than their rigid counterparts^23^. In an effort to simplify our subsea control system and produce a scalable manipulator, we created a hybrid design consisting of soft modules that produce well-defined motions about a single axis. The gripping module is based on previous work experimenting with various finger designs and configurations, and all other components are based on soft actuator modules that have been demonstrated to function under extreme hydrostatic pressures. The rotary and bending modular actuators have been previously characterized extensively using both pneumatic and hydraulic pressure^24^, and several soft gripping modules have also been described^10,13^.

**Figure 3:**
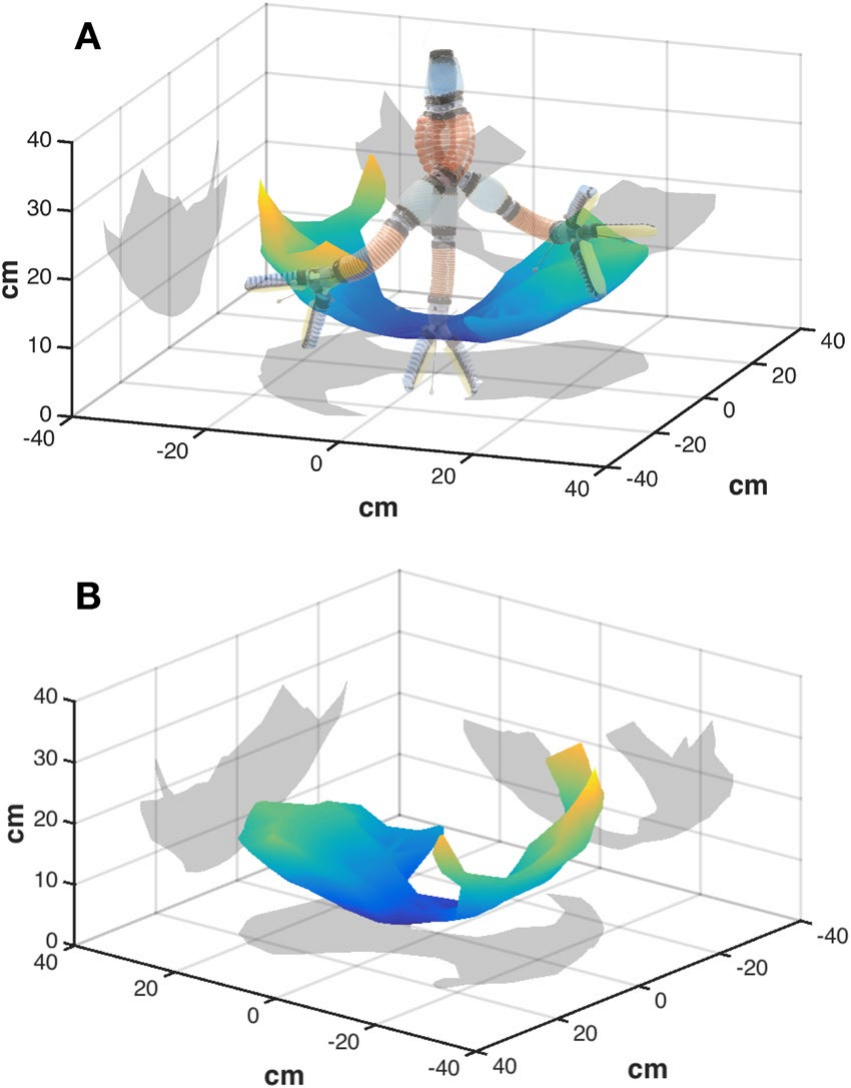
(**A**) Workspace of a five-module soft arm, mechanically grounded at the uppermost vertical position. Rotary modules in the arm are shown in blue, bending in red, and a three-finger gripping module is mounted at the distal end. (**B**) The same workspace, viewed at a different perspective.

Rotating, bending and gripping modules are added or removed to achieve a desired workspace, payload, and size. Figure 3 shows a representative workspace analysis for a five-module system, oriented vertically to counter gravity effects experienced in the motion capture laboratory. In this configuration the arm can reach greater than 50 cm side-to-side with approximately 25 cm of vertical positioning. Each module has almost negligible weight in water, allowing for a wide range of mounting orientations on a subsea vehicle. The arm can be configured redundantly to achieve various approach angles from the gripper, and faulty modules can be replaced quickly without removing the entire arm from the deployment platform.

### Open-circuit seawater hydraulic engine

The modular soft manipulator we present here requires proportional, multi-port control of low-pressure hydraulics to facilitate delicate actuation. While a variety of hydraulic fluids are compatible with our actuators, seawater was chosen for the following reasons: (1) environmental considerations due to leakage; (2) the ability to draw from/vent to zero-differential (ambient) pressure without introducing a foreign fluid to a pristine environment; and (3) the relatively low viscosity of seawater. Despite these advantages, seawater is highly corrosive to ferrous metals and aluminum. Therefore, components of the hydraulic engine were chosen for both electrohydraulic performance and their resistance to corrosion. The open-circuit design allows fresh seawater to circulate through the system, which can be completely flushed out with freshwater and dry air following a dive. While seawater hydraulics has been demonstrated in the past^25^, these high-pressure, high-flow systems require extensive filtering to remove particulates from the fluid. The minimal amount of fluid required to actuate soft modules affords the advantage of reduced filtration needs, while soft actuators themselves are unaffected by particulate matter.

**Figure 4:**
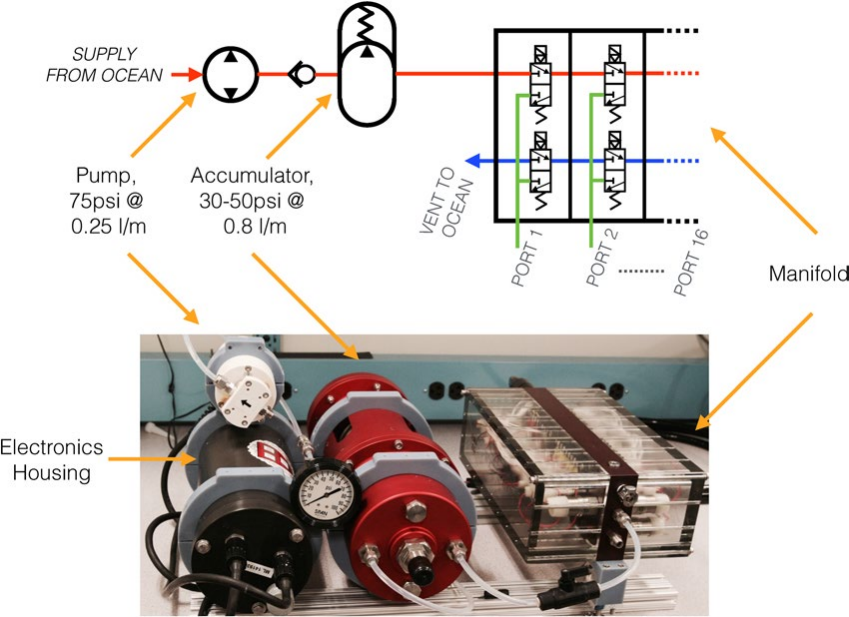
Hydraulic schematic and image of the open-circuit seawater hydraulic engine. The electronics housing contains both pump and valve control components.

The hydraulic engine is composed of three modules: a pump, a hydraulic accumulator, and a valve manifold (Fig. 4). A fixed displacement deep-sea pump was chosen for its ability to pump seawater at a high pressure (75 psi at 250 ml/min) within a compact form factor. To increase the flow available to the manifold, a custom hydraulic accumulator was integrated to store up to 1.5 liters of seawater maintained at 30–50 psi. This provides constant hydraulic pressure to a custom hydraulic manifold populated with 32 miniature stainless steel proportional valves, designed in a similar fashion to existing pressure-compensated hydraulic systems^26^. The manifold can accommodate up to 16 actuation ports, each allowing for proportional control of pressure supply and relief. The exact number of ports required is dependent on the combination of modules chosen; a bending module requires two actuation ports, while the rotary and gripping modules each require a single port to actuate.

**Figure 5:**
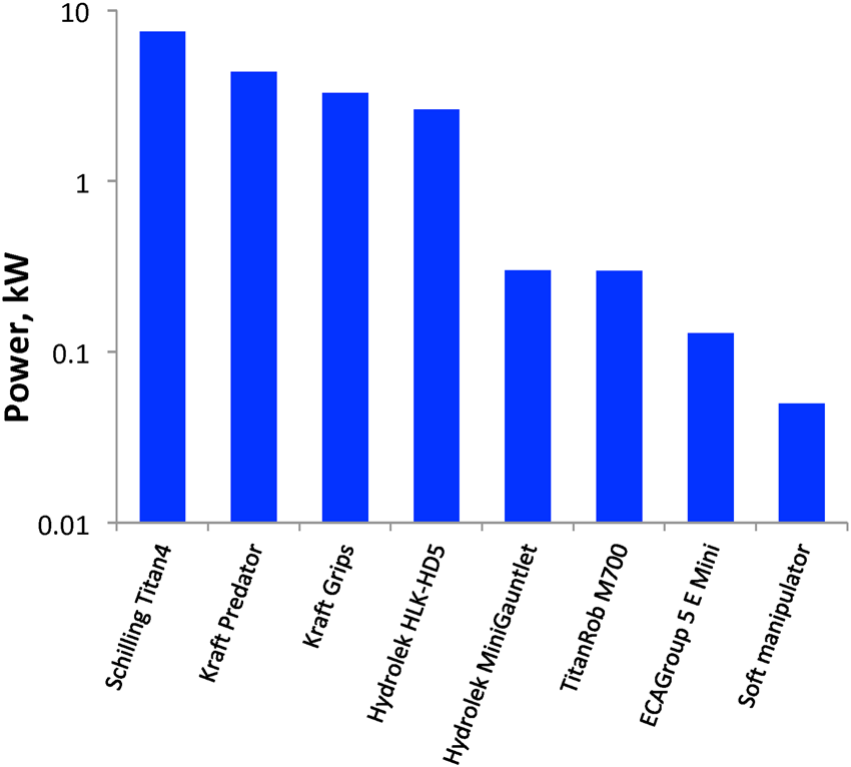
Logarithmic power comparison of several leading deep-sea manipulator systems. All are actuated hydraulically with the exception of ECAGroups’ 5 E Mini (www.ecagroup.com), which uses electronic linear and rotary actuators. The HydroLek MiniGauntlet (www.hydro-lek.com) is considered the industry standard small form-factor hydraulic manipulator, typically used on lightweight ROV’s that are limited in the power and payload they can provide for manipulation.

Our seawater hydraulic engine requires minimal power to operate, due to the low pressure differential produced from the surrounding environment and the nominal fluid volume required to actuate the soft arm. Unlike conventional hydraulic systems, which constantly run a pump while regulating pressure in relation to system demand, the design presented here delivers pressure and flow on-demand to afford increased efficiency and fast response times. When active, the pump requires approximately 12 W and each valve draws a maximum of 7.2 W, resulting in a maximum power budget of ~41 W when four ports are active (as shown on the submersible in Fig. 1) and ~62 W when seven ports are active (as shown in Fig. 3). In practice, the average power required to operate is much lower. Furthermore, the accumulator allows the soft arm to operate without the pump running for short periods of time. Our hydraulic system requires less than half of the power of the smallest commercially available deep-sea electronic manipulator, and approximately one-sixth of the power required by a standard small form factor hydraulic manipulator (Fig. 5).

### Control system

**Figure 6:**
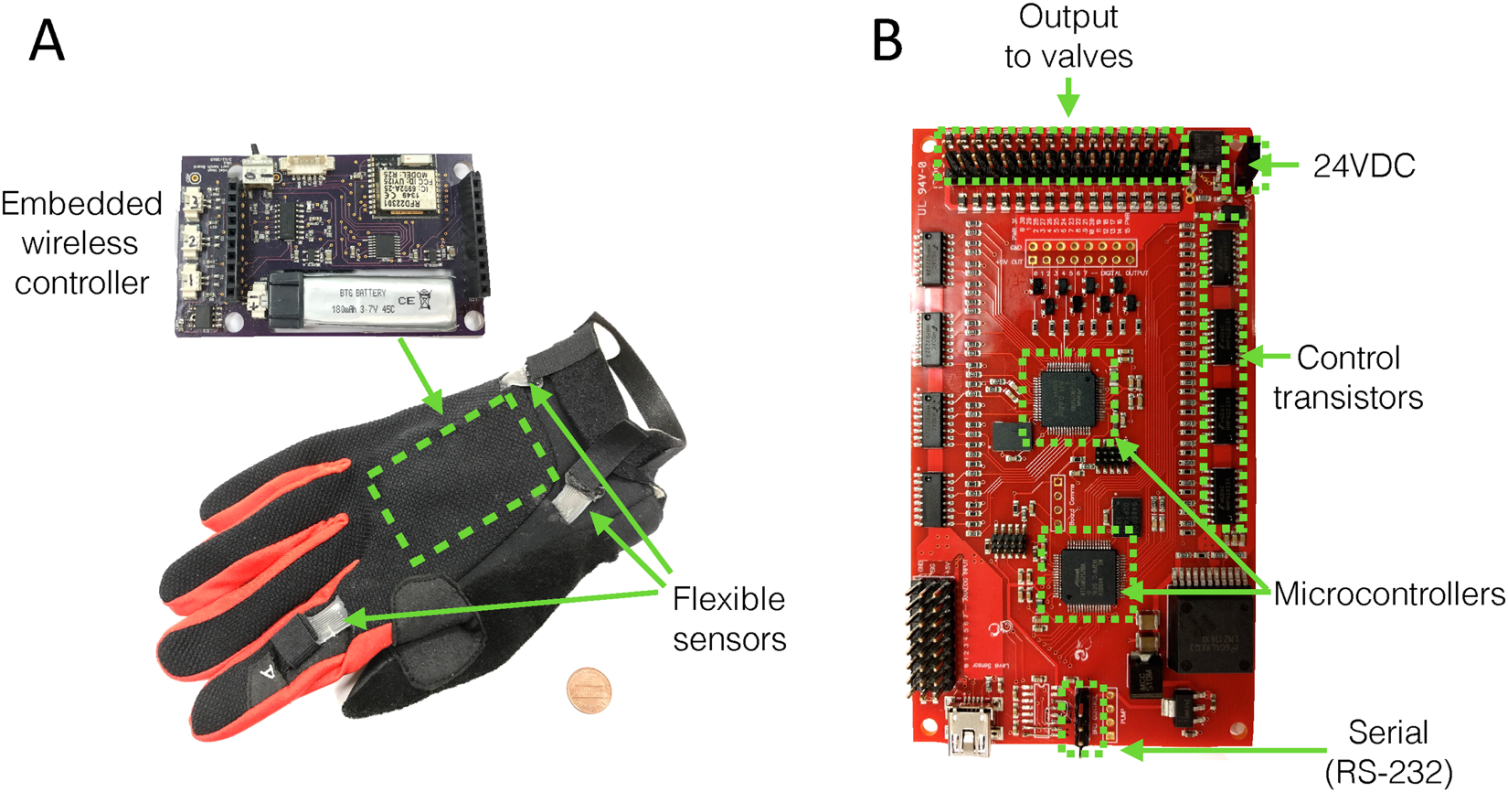
(**A**) Images of wireless glove controller; the reader is referred to Vogt & Wood^17^ for a detailed description of this system. (**B**) Subsea valve controller board, which receives serial commands from the glove controller’s topside software. The board is based on dual 32-bit ARM microcontrollers, which send 0–24VDC output signals directly to each proportional valve.

Since ROVs and manned submersibles provide real-time video or have human observers present, a visual-based open-loop control system was chosen to operate the soft manipulator. Individual valves in the manifold are actuated using a custom PWM control board contained inside a deep-sea housing along with the control electronics for the pump. Proportional 8-bit resolution of each port, which consists of two valves (pressure and relief), offers a total of 128 incremental settings for each valve. Remote telemetry to both units is achieved via RS-232 serial communication, along with a single 24VDC power source. A human operator uses a wireless glove equipped with flexible sensors, which sends serial commands to a subsea valve control board (Fig. 6). The glove interface translates intuitive hand motions into discrete actuations; gripping is achieved by curling the index finger, while wrist gestures control bending and rotating motions in the manipulator. A custom topside GUI allows for glove calibration and direct control of individual valves.

### Field testing

A proof-of-concept system using only the McLane pump system and a soft gripper was deployed on the ROV *HERCULES* during an expedition to the Channel Islands National Marine Sanctuary in July 2016. A Predator manipulator (Kraft Telerobotics, Overland Park, KS USA) was used to position the gripper on the seafloor. The modular gripper was successfully operated at depths exceeding 1800 m (2700 psi) and was used to collect deep-sea organisms including a slime star, glass sponge, and an extremely delicate holothurian (Fig. 2D). In these field trials, there was an occasional loss of a gripping actuator at its mounting point, a problem addressed in subsequent designs. The complete hydraulic engine was then tested to approx. 2300 m (3500 psi) in a pressure test facility (Woods Hole Oceanographic Institution, Woods Hole, MA USA). A camera installed inside the pressure vessel provided live visual feedback while various soft actuators were operated.

**Figure 7:**
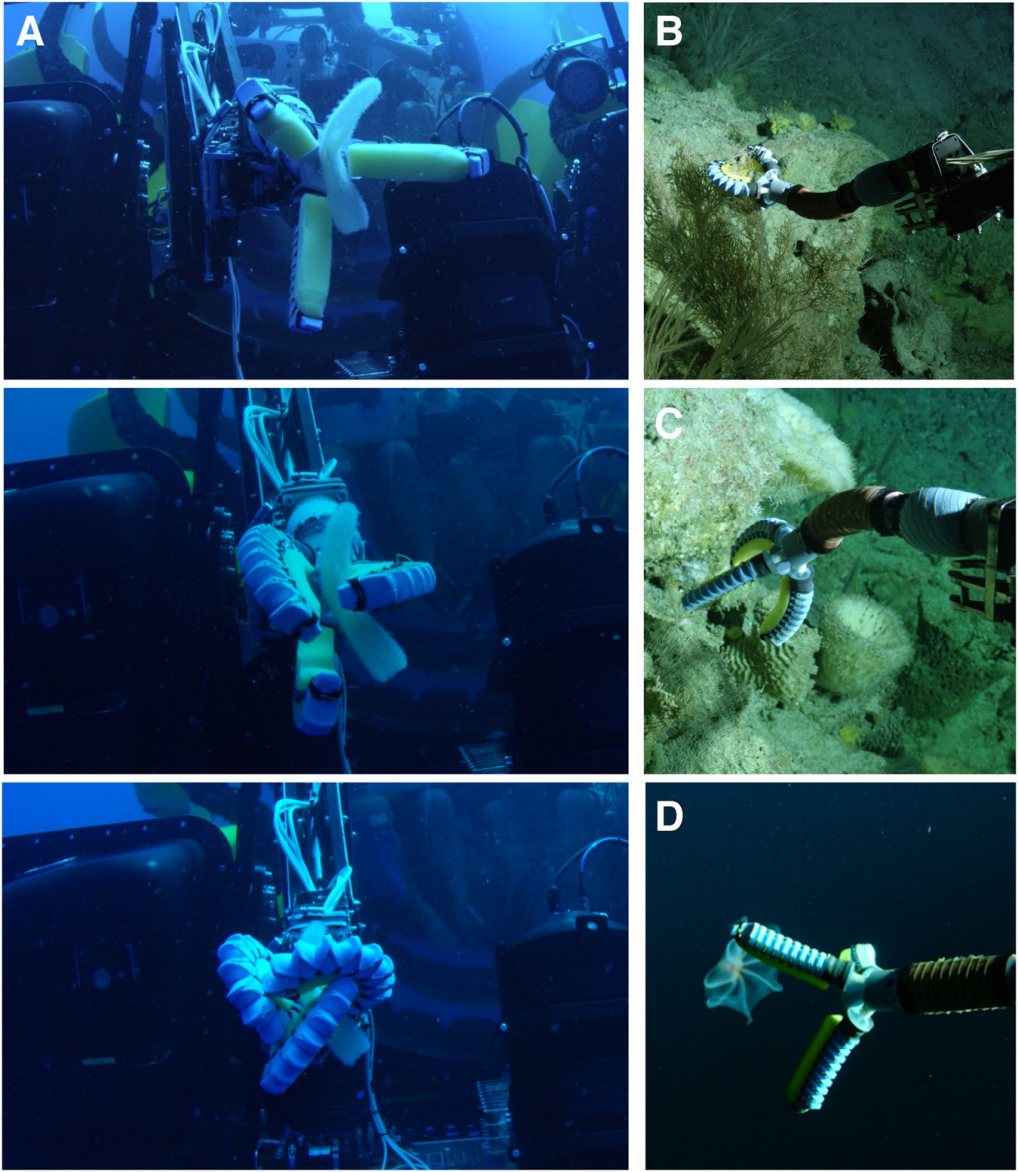
Field testing in the Fernando de Noronha Archipelago, Brazil using a Triton 3K3 manned submersible. (**A**) Image sequence of the soft manipulator grasping a midwater pyrosome (*Pyrosoma atlanticum*) in the water column, as observed from a diver. (**B**) The soft manipulator articulating upwards to grasp a coral at approximately 300 m depth and (**C**) downwards to grasp a sponge. (**D**) Manipulator straightened out to approach the deep-sea octopus *Cirroteuthidae murrayi* at approximately 700 m. Images are taken from frames of a Red Digital 8 K Dragon pan-tilt camera mounted to the outside of the submersible.

The complete soft manipulator and hydraulic engine was deployed onboard a Triton 3300/3 manned submersible during an expedition to explore mesophotic coral reefs in the Fernando de Noronha Archipelago, Brazil. The soft manipulator was attached to the wrist of an HLK-CRA6 arm (Hydro-Lek, Hampshire UK) and was used to grasp specimens including a branching coral, a midwater pyrosome (*Pyrosoma atlanticum*), and several other animals at depths exceeding 700 m (Fig. 7). During these field tests, the bending and rotary modules were accidently subjected to higher differential pressures than they were designed for (40–50 psi, design pressure is 0–20 psi) and the modules survived these occasional overpressure events. The biggest challenge encountered was securing the entire manipulator during submersible launch and recovery, where wave motion subjected the system to considerable water currents. The modules did survive launch and recovery, but occasionally required re-sealing using silicone glue prior to redeployment. Because manned submersibles have a limited battery capacity, the minimal power requirements of the seawater hydraulic engine offered an operational advantage over the existing manipulator system; submersible pilots reported that they could only run their industrial-class arm for a few minutes at a time, while the soft arm was not a consideration for operational endurance.

## Discussion

Several examples exist of multi-DOF soft manipulators, including bioinspired continuum octopus-like arms^15,27^, closed-loop control of 2D continuum arms^28^, and inflatable actuator arms^29,30^. These systems demonstrate a range of advantages associated with using compliant materials to achieve multi-DOF actuation, but are rarely validated in a practical setting. The soft manipulator we present here is notably distinguished from previous efforts through its use of hydraulic pressure to achieve robust actuation, a consolidated control system for deployment in remote and harsh environments, and its use of modular actuators that can be easily added or removed. Furthermore, it has been demonstrated at depth to grasp and collect delicate specimens, a long-standing challenge for deep-sea biologists.

Soft actuators are typically controlled pneumatically, owing to the availability of a wide variety of off-the-shelf control components (e.g. pumps and valves) and the convenience of a dry development environment. Gases dramatically compress in volume under high hydrostatic pressures, and so these advantages are not afforded in the deep ocean. Since all commercially available subsea hydraulic systems operate at high pressures exceeding 1000 psi that are incompatible with soft actuators, an entirely new hydraulic control system was created. The open-circuit seawater hydraulic engine we present in this paper is based on existing principles established for heavy-duty deep sea hydraulic systems, but instead delivers the precision control of low-pressure fluids that soft actuation requires. Its compatibility with seawater presents additional practical advantages, particularly for plastic and silicone-derived soft actuators that are not subject to galvanic corrosion. The low power requirements of the seawater hydraulic engine make it suitable for vehicles with tightly constrained power budgets, while future efforts to incorporate closed-loop control would facilitate autonomous manipulation, a growing trend in marine robotics^31,32^. Equipping autonomous underwater vehicles (AUV’s) for physical sampling takes advantage of their sophisticated survey and navigational capabilities^33^, allowing for more efficient exploration and experimentation.

While the soft manipulator presented here is intended to replace a rigid arm, the modularity of our system allows for existing manipulators to choose between various tools and actuators *in situ*. Rotary and bending modules can be sequentially added or removed to tune the workspace of the manipulator, and are easily replaced in case of failure. In our field tests, we found the two-actuator wrist to be the maximum practical length that could survive launch and recovery of the deep-sea platform. This limitation could be addressed in several ways including increasing the overall stiffness of the wrist actuators, utilizing a removable protective cover, or deploying while in a coiled or otherwise protected state. Such considerations are not normally encountered in a laboratory setting, highlighting the need to incorporate field-testing in development efforts.

This work describes the first stand-alone soft manipulator designed for use in the marine environment. The hydraulic engine provides proportional control on 16 channels, requires minimal power to operate, and has been demonstrated remotely at depths exceeding 2300 m. The manipulator is composed of separate soft actuators with discrete ranges of motion, which are combined to achieve a practical and adjustable workspace. Our goal is to improve robustness and incorporate spatial and force feedback, allowing for haptic and autonomous control of an inherently gentle and underactuated system. We are also exploring incorporating non-invasive *in situ* genomic preservation capabilities (RNA, DNA and eDNA) into the actuators that utilize aspects of this soft robotic arm system.

## Methods

### Actuators

Gripping actuators were constructed using methods described by Galloway *et al*.^10^ and further refined based on field tests. Rotating and bending modules were constructed using methods described by Kurumaya *et al*.^24^. Compatible modular connectors were produced using a photopolymer 3D printer (Stratasys Objet Connex500), and a variety of quick-disconnect fittings were employed to connect hydraulic lines.

### Hydraulics and controls

The open-circuit seawater hydraulic engine was comprised of several components (Fig. 4). The deep-sea pump was a 250 ml/min model originally designed for use in a phytoplankton sampler (McLane Phytoplankton Sampler, McLane Research Laboratories, East Falmouth, MA USA). The custom 1.5 liter 30–50 psi hydraulic accumulator was produced by KystDesign Sub-Sea Technology (Aksdal, Norway). The hydraulic manifold was populated with 32 miniature stainless steel proportional valves designed for use with corrosive fluids (model #PFV-W24-M100C-0400, Enfield Technologies, Shelton, CT USA). Control of the valves was provided by a custom circuit board based on dual Atmel SAML21J18b 32-bit ARM microcontrollers. Both the pump and valve control boards were housed inside a 3000 m-rated 1 atm housing with external 24VDC power.

### System evalulation

Ten Vicon T-Series cameras were used to capture the workspace of the soft arm. Four reflective markers were attached to the end effector and tracked through the Vicon Tracker software and a Matlab xPC system to determine the position and orientation. The outer boundary of the collected data was then extracted and used to determine the maximum achievable workspace. Pressure testing of the actuators and hydraulic system was conducted at the Woods Hole Oceanographic Pressure Test Facility (https://www.whoi.edu/page.do?pid=10359), with iterative tests at 500, 1000, 1500, 2000, and 2300 m. Field tests of the soft gripper were conducted onboard the E/V *Nautilus* using the ROV *Hercules* in June 2016. The complete soft arm was tested in May 2017 onboard the M/Y *Alucia* using the Triton 3K3 manned submersible *Nadir*. Additional gripper testing was conducted onboard the R/V *Falkor* using the ROV *SuBastian* in October 2017.
